# New Books

**Published:** 2006-12

A Primer on Environonmental Decision-Making: An Integrative Quantitative Approach

Knut Lehre Seip, Fred Wenstøp

New York:Springer, 2006. 496 pp. ISBN: 1-4020-4073-3, $149

Advanced Analysis of Gene Expression Microarray Data

Aldong Zhang

Hackensack, NJ:World Scientific Publishing Co., 2006. 356 pp. ISBN: 981-256-645-7, $68

Advances in Molecular Toxicology

James C. Fishbein

Burlington, MA:Elsevier, 2006. 200 pp. ISBN: 0-444-52842-3, $200

Alternatives to Animal Testing

R. M. Harrison, R. E. Hester, eds.

New York:Springer, 2006. 130 pp. ISBN: 0-85404-211-3, $89.95

Basic Methods for the Biochemical Lab

Martin Holtzhauer

New York:Springer, 2006. 252 pp. ISBN: 3-540-32785-1, $69.95

Beyond Limits? Dealing with Chemical Risks at Work in Europe

David Walters, Karola Grodzki

Burlington, MA:Elsevier, 2006. 430 pp. ISBN: 0-08-044858-5, $99.95

Communicating Science

Pierre Laszlo

New York:Springer, 2006. 214 pp. ISBN: 3-540-31919-0, $29.95

Environmental and Pollution Science

Ian Pepper, Charles Gerba, Mark Brusseau

Burlington, MA:Elsevier, 2006. 552 pp. ISBN: 0-12-551503-0, $89.95

Environmental Toxicology

A. G. Kungolos, C. A. Brebbia, C. P. Samaras, V. Popov, eds.

Billerica MA:WIT Press, 2006. 384 pp. ISBN: 1-84564-045-4, $225

Global Climate Change and Response of Carbon Cycle in the Equatorial Pacific and Indian Oceans and Adjacent Landmasses

Hodaka Kawahata, Yoshio Awaya, eds.

Burlington, MA:Elsevier, 2006. 450 pp. ISBN: 0-444-52948-9, $185

Global Environmental Assessments: Information and Influence

Ronald B. Mitchell, William C. Clark, David W. Cash, Nancy M. Dickson, eds.

Cambridge, MA:MIT Press, 2006. 352 pp. ISBN: 0-262-63336-1, $27

Government and Research: Thirty Years of Evolution

Maurice Kogan, Mary Henkel, Steve Hanney

New York:Springer, 2006. 247 pp. ISBN: 1-4020-4444-5, $119

Inorganic and Organic Lead Compounds

International Agency for Research on Cancer

Geneva:World Health Organization Press, 2006. 519 pp. ISBN: 928-321-287-8, $49.50

Lives Per Gallon: The True Cost of Our Oil Addiction

Terry Tamminen

Washington, DC:Island Press, 2006. 272 pp. ISBN: 1-59726-101-7, $24.95

Long-Term Ecological Change in the Northern Gulf of Alaska

R. B. Spies

Burlington, MA:Elsevier, 2006. ISBN: 0-444-52960-8, $99

Survival Skills for Scientists

Federico Rosei, Tudor Johnston

Hackensack, NJ:World Scientific Publishing Co., 2006. 200 pp. ISBN: 1-86094-640-2, $38

The Future of Sustainability

Marco Keiner, ed.

New York:Springer, 2006. 257 pp. ISBN: 1-4020-4734-7, $80

The Global Genome: Biotechnology, Politics, and Culture

Eugene Thacker

Cambridge, MA:MIT Press, 2006. 464 pp. ISBN: 0-262-70116-2, $19.95

